# Correction: A novel method for the highly efficient biotransformation of genistein from genistin using a high-speed counter-current chromatography bioreactor

**DOI:** 10.1039/c9ra90015b

**Published:** 2019-03-05

**Authors:** Daijie Wang, Muhammad Shafiq Khan, Li Cui, Xiangyun Song, Heng Zhu, Tianyu Ma, Xiaoyu Li, Rong Sun

**Affiliations:** Key Laboratory of TCM Quality Control, Shandong Analysis and Test Center, Qilu University of Technology (Shandong Academy of Sciences) Jinan 250014 P. R. China; Department of Biotechnology and Bioinformatics, International Islamic University Islamabad 44000 Islamic Republic of Pakistan; College of Pharmacy, Shandong University of Traditional Chinese Medicine Jinan China; Institute of Advanced Medical Science, Shandong University Jinan 250012 P. R. China sunrong@sdu.edu.cn; The Second Hospital of Shandong University Jinan 250033 P. R. China

## Abstract

Correction for ‘A novel method for the highly efficient biotransformation of genistein from genistin using a high-speed counter-current chromatography bioreactor’ by Daijie Wang *et al.*, *RSC Adv.*, 2019, **9**, 4892–4899.

The authors regret that an incorrect affiliation was included in the original article. The correct affiliations are as presented above. The following funding acknowledgement was also omitted: “Traditional Chinese Medicine Pharmacology and Toxicology Expert (NO.ts201511107) from Taishan Scholar Project of Shandong Province”.

The Royal Society of Chemistry apologises for these errors and any consequent inconvenience to authors and readers.

## Supplementary Material

